# Why more research needs to be done on echinococcosis in Pakistan

**DOI:** 10.1186/s40249-017-0309-z

**Published:** 2017-07-03

**Authors:** Haroon Ahmed, Shahzad Ali, Muhammad Sohail Afzal, Abid Ali Khan, Hamid Raza, Zaheer Hussain Shah, Sami Simsek

**Affiliations:** 1 Department of Biosciences, COMSATS Institute of Information Technology (CIIT), Park Road, Chak Shahzad, Islamabad Pakistan; 2 Department of Parasitology, Faculty of Veterinary Medicine, University of Firat, 23119, Elazig, Turkey; 3 Department of Wildlife and Ecology, University of Veterinary and Animal Sciences, Lahore, Pakistan; 4 Department of Chemistry, School of Science, University of Management and Technology (UMT), Lahore, Pakistan; 5 School of Science, University of Management and Technology (UMT), Lahore, Pakistan

**Keywords:** Pakistan, Echinococcosis, Neglected tropical diseases, Research

## Abstract

**Background:**

Echinococcosis has a worldwide geographical distribution with endemic foci on every inhabited continent. Due to the frequent outbreaks in different parts of Pakistan in the recent past, echinococcosis is being described as a neglected tropical disease and is considered one of the most neglected parasitic diseases in the country. In endemic regions, predominantly settings with limited resources, there are high numbers of echinococcosis patients, as these communities do not have access to appropriate treatment. In Pakistan, there are limited reports on echinococcosis. The disease is prevalent in human and livestock, but this has not been sufficiently explored yet. Pakistan is an agricultural country and due to the disease’s zoonotic mode of transmission, there is a dire need of future research on it. The present paper is an effort to highlight the importance of echinococcosis in Pakistan.

**Discussion:**

There is a dire need for future research on echinococcosis in Pakistan as very few investigations had been carried out on this topic thus far. The prevalence of the disease in neighbouring countries highlights that Pakistan might be at severe risk of this zoonotic infection and further supports the need for more research. In Pakistan, the majority of the population lives in rural areas with limited acess to proper hygienic/sanitary facilities. These conditions favour the outbreak of diseases such as echinococcosis. The limited available data could result in higher outbreaks in the future, and thus cause the already weak healthcare system to overburden.

The country has a meagre annual budget for health, which is being spent on known infections such as polio, dengue fever and hepatic viral infections. A proper surveillance system for echinococcosis is required across the country as treatment is usually expensive, complicated and may require extensive surgery and/or prolonged drug therapy. Development of new/novel drugs and other treatment modalities receives very little, if any, attention. Prognostic awareness programmes against this infection involve deworming of the infected animals, improved food inspection and slaughterhouse hygiene, and public education campaigns.

**Conclusion:**

Future research on echinococcosis is anticipated to demonstrate whether the epidemiology, diagnosis and recombinant vaccines/antibodies relating to echinococcosis can meet the quality standards (purity, potency, safety and efficacy) defined by the World Health Organization. Research work should be carried out on the epidemiology and serodiagnosis of echinocossis in the different areas of Pakistan, which will be useful for the proper eradication of echinococcosis in this region. The health department should implement awareness-raising campaigns for the general public in order to reduce the burden of disease.

**Electronic supplementary material:**

The online version of this article (doi:10.1186/s40249-017-0309-z) contains supplementary material, which is available to authorized users.

## Multilingual abstracts

Please see Additional file 1 translations of the abstract into the five official working languages of the United Nations.

## Introduction

Echinococcosis is a human and animal health problem in many endemic areas worldwide. It is considered a neglected zoonotic disease caused by the larval form of the tapeworm *Echinococcus* spp*.* Humans can become infected through the accidental intake of parasitic eggs excreted by the faeces of definitive hosts such as dogs, foxes and other canids [1]. Cystic echinococcosis (CE) is a chronic, complex and still neglected tropic disease (NTD) in many countries [2].

At least 270 million people (58% of the total population) are at risk of acquiring CE in Central Asia, including in areas of Mongolia, Kazakhstan, Kyrgyzstan, Tajikistan, Turkmenistan, Uzbekistan, Afghanistan, Iran, Pakistan and Western China [3]. The World Health Organization (WHO) included CE in a subgroup of selected NTDs to be addressed within its 2008–2015 strategic plan to control NTDs [4, 5]. In September 2016, the WHO further expanded its network of collaborating centers for echinococcosis. The newest two centres in Xinjiang, China and in Rome, Italy will help sustain control strategies and the implementation of uniform operational procedures, testing and training.

The WHO recommends that the impact of zoonotic infections be assessed before any control measure is implemented [6, 7]. According to the latest WHO report, at any time, more than one million people are infected with echinococcosis across the world. The WHO is working towards the validation of effective CE control strategies by 2020 [8].

Neglected tropical diseases are a diverse group of communicable diseases that prevail in tropical and subtropical conditions in 149 countries and affect more than one billion people, costing developing economies billions of dollars every year. These diseases mainly affect populations living in poverty, without adequate sanitation and which are in close contact with infectious vectors and domestic animals and livestock. At least one seventh of the world’s population is affected by NTDs, including roughly 800 million children.

In Pakistan, the outbreaks of dengue – declared to be the world’s ‘fastest-spreading NTD’ by the WHO in January 2013 – in 2010 and 2011 were a stark illustration of the challenges which persist in this realm of global health. Traditionally, NTDs exist exclusively in the poorest and most marginalised communities, receiving little attention from the international health community, despite the fact that they are the most common infections among the world’s poorest. The NTDs being targeted by the reports are lymphatic filariasis, soil-transmitted helminthiasis, schistosomiasis, echinococcosis, onchocerciasis, visceral leishmaniasis, trachoma, leprosy, guinea worm, chagas disease and human African trypanosomiasis.

In Pakistan, echinococcosis is one of the most important NTDs and the present letter was written to highlight the importance of echinococcosis among humans and livestock in the country.

## Discussion

Pakistan is a country with a low socio-economic status. It is highly populated with approximately 200 million inhabitants, with most of these living in rural areas or very crowded urban areas with poor sanitary facilities. A large proportion of Pakistanis is affiliated with agriculture and local dairy farming on a small scale. The workers on these small farms come into close contact with animals and since proper health and hygiene principles are not strictly followed, the inhabitants of these areas are also at high risk of acquiring *Echinococcus* spp*.* infections.

Humans can become infected through ingestion of parasite eggs in contaminated food, water or soil, or via direct contact with animal hosts. It has been shown that common sheep (G1) and buffalo (G3) strains of *E. granulosus* are circulating among livestock in Punjab and that these strains are highly adaptable to goats, camels and cattle. The molecular characterization of human cysts infected with *Echinococcus* spp. belong to the common sheep strain (G1) of *E. granulosus*, reinforcing the fact that this strain has the potential for zoonotic transfer. Both morphological and molecular characterisations of *Echinococcus* spp. in Pakistan support similar findings from other parts of the world, suggesting that *Echinococcus* of sheep and buffalo origin is phenotypically and genetically similar worldwide. This adds further evidence to support its recognition as one species, namely *E. granulosus* sensu stricto [9].

Echinococcosis is a NTD and few investigations have been carried out on its prevalence in Pakistan. However, the limited literature that is available from Sindh, Punjab and Khyber Pakhtunkhw highlights the prevalence of the disease in the Pakistani population [10–15]. Echinococcosis infection is also reported in humans from neighbouring countries of Pakistan such as India, China, Iran, Afghanistan and Occupied Kashmir [16–32]. The reported prevalence of echinococcosis in different areas of Pakistan between 1989 and 2015 was between 2.44 and 35% in cattle [9, 14, 33–35], between 7.19 and 24.40% in buffaloes [9, 36, 37], between 3.24 and 8.85% in sheep [9, 35, 37, 38], between 2.44 and 6.61% in goats [9, 35, 38], and 17.29% in camels [9].

In Pakistan, there is a lack of research and data on echinococcosis and so future research on this infection is indispensable. The prevalence of echinococcosis in neighbouring countries highlights that this zoonotic infection may become endemic in Pakistan and further supports the need for more research. A major proportion of the population lives in rural areas with poor health hygienic/sanitary facilities, which further concretes the aforementioned hypothesis. Even though the WHO has declared the disease as a NTD, few published reports are available on the epidemiology of the disease in humans and animals. Research on the distribution pattern, intensity and molecular characterisation should be carried out in the near future in the different endemic areas of Pakistan.

The lack of information on this disease might put the Pakistani population at high risk and might result in a future burden for the country’s health system. Furthermore, there is no health insurance system in Pakistan to support infected individuals. A proper monitoring system for echinococcosis is required across the country as treatment is often expensive, complicated and may require extensive surgery and/or prolonged drug therapy. Some sort of eradication strategies should be implemented on the national level to reduce the burden of the disease. Keeping these in mind, below is a summary of the questions/considerations that should be addressed on a priority basis.

### Why are there limited studies on echinococcosis in Pakistan?

Echinococcosis has been declared as a NTD in Pakistan. Why are there not any comprehensive studies on the topic? There are a limited number of parasitologists, especially helminthologists, in Pakistan and most have turned a deaf ear to the importance of echinococcosis because data collection is difficult, whether in the hospital or in the field. Most of the specialists are solely focused on diseases such as dengue and other viral diseases, which might be due to the higher prevalence rates of these diseases and more funding resources allocated to them.

### The WHO declared echinococcosis as a NTD even though there is a lack of research on it

Until now, only eight reports on echinococcosis in livestock [9, 30, 33–38] and one doctoral thesis [39] have been published in Pakistan. To the best of our knowledge, from 1980 to 2015, only three reports based on prevalence data and 13 case reports on echinococcosis in humans in different body regions (abdomen, ovary, cystic, pelvic, renal, cervical, femoral, breast, pneumothorax, brain [cerebral] and urinary) have been published in Pakistan [10–14, 40–50]. There is no comprehensive study on molecular epidemiology and genetic characterisation of the disease. Therefore, our aim is to make the scientific community/researchers explore echinococcosis in Pakistan. Our research group had submitted various proposals on echinococcosis in Pakistan to national and international funding agencies.

### Scientists should explore the disease in the different agro-climatic regions of Pakistan

There are limited scientific reports from Pakistan on this topic and the majority of studies focus on epidemic diseases such as dengue. Since very meagre work has been done on this parasitic disease, we hope that this letter would be read by many scientists through your esteemed journal. This letter would hopefully facilitate us in the awarding of funding from national/international organisations for carrying out research on echinococcosis in Pakistan.

### What are the different risk factors for echinococcosis in the different endemic areas of Pakistan?

According to recent estimates, the geographical distribution of *Echinococcus* spp. infection is expanding, and becoming an emerging and re-emerging problem in several regions of the world. Echinococcosis endemicity is geographically heterogeneous and over time may be affected by global environmental change. Therefore, landscape epidemiology offers a unique opportunity to quantify and predict the ecological risk of infection at multiple spatial and temporal scales. The most relevant environmental sources of spatial variation in human echinococcosis risk, and the potential applications of landscape epidemiological studies to characterise the current patterns of parasite transmission across natural and human-altered landscapes has been investigated [51].

Pakistan has a rural population and livestock rearing is a common practice. Each family owns one or two livestock animals as well as a pet dog. Vegetables and fruit, which have not been washed with clean water, are sold in markets. People consume vegetables and fruit contaminated with echinococcus eggs, and have no information about this disease and its associated risk factors.

Researchers have reported that the major factors contributing to the onset of this disease are an abundance of dogs, engagement in risky behaviour and poor abattoir infrastructure and practices [52]. Others have predicted that, in China, age, gender, herding population, farming population, nomadic populations, education level, income level, livestock ownership, dogs, vole density, deforestation and overgrazing all contribute to the onset of the disease [53]. Our research team will be submitting new projects on the prevalence, genotyping and risks factors associated with echinococcosis in Pakistan to various national and international organisations to explore echinococcosis in Pakistan.

### Genotyping and molecular characterization of echinococcosis in Pakistan

In Pakistan, only two research papers have been published on genotyping and molecular characterization of echinococcosis [9, 14]. Researchers should collect samples from all over Pakistan and then perform characterisation to determine the strains in the country.

### There is no enough endemic or disease burden data from Pakistan to support the research priorities of echinococcosis

As there is not enough data on this disease and it is not explored sufficiently in Pakistan, very little is known about the exact prevalence of the disease in the different regions of the country.

Globally, cestode zoonoses cause serious public health problems, particularly in Asia. Among all neglected zoonotic diseases, cestode zoonoses account for over 75% of global disability-adjusted life years (DALYs) lost. An international symposium on cestode zoonoses research and control was organised in China on 28–30 October 2013 to establish joint efforts to research effective approaches to control these zoonoses. It brought together 96 scientists from the Asian region and beyond to exchange ideas, report on progress, make a gap analysis and distil priority settings with a focus on the South Asian region. Key objectives were to agree on solutions to accelerate progress towards decreasing transmission, and human mortality and morbidity caused by the three major cestode zoonoses (CE, alveolar echinococcosis and cysticercosis); to critically assess the potential to control these diseases; to establish a research and validation agenda on existing and new approaches; and to report on novel tools for the study and control of cestode zoonoses [54].

There is a dire need for cooperation between Central Asia countries in the control of hydatid cyst disease. Meetings on echinococcosis in Asia should be organised on a regular basis to highlight the incidence and burden of echinococcosis. On the basis of these, hot spot area s should be identified and control strategies should be implemented.

## Conclusion

Future research on echinococcosis is anticipated to demonstrate whether the epidemiology, diagnosis and recombinant vaccines/antibodies relating to echinococcosis can meet the quality standards (purity, potency, safety and efficacy) defined by the WHO. Research work should be carried out on epidemiology and serodiagnosis of echinocossis in the different areas of Pakistan, which will be useful for the proper eradication of echinococcosis from this region. The health department should implemente awareness-raising compagins for the general public in order to reduce the burden of disease.

## Additional file

Additional file 1: Multilingual abstracts in the five official working languages of the United Nations. (PDF 633 kb)

## Abbreviations

CE: Cystic echinococcosis
NTD: Neglected tropical disease
WHO: World Health Organization
