# Supplementary material for: Why more research needs to be done on echinococcosis in Pakistan
Source: Infect Dis Poverty. 2017 Jul 3;6:90. doi: 10.1186/s40249-017-0309-z (PMC5494903; doi:10.1186/s40249-017-0309-z)
Supplement: Additional file 1: — Multilingual abstracts in the five official working languages of the United Nations. (PDF 633 kb) [file 40249_2017_309_MOESM1_ESM.pdf]

Translation of the abstract into the six official working languages of the United Nations

لماذا هناك حاجة إلى القيام بمزيد من البحوث على مرض الأكياس المائية في باكستان؟

هارون أحمد، شاه زاد علي، محمد سهيل أفضال، عبد علي خان، حميد رضا، زهير حسين شاه، سامي سيمسك

الملخص

الخلفية: لمرض الأكياس المائية (أو داء المشوكات) توزيعاً جغرافياً في جميع أنحاء العالم مع بؤر مستوطنة في كل قارة مأهولة. ونظراً للتفشي المتكرر في أجزاء مختلفة من باكستان في الماضي القريب، يتم وصف داء المشوكات باعتباره من أمراض المناطق المدارية المهملة، ويعتبر واحداً من الأمراض الطفيلية الأكثر إهمالاً في البلاد. في المناطق الموبوءة، الأوضاع في الغالب ذات موارد محدودة، وهناك أعداد كبيرة من مرضى الأكياس المائية ، حيث أن هذه المجتمعات لا تحصل على العلاج الملائم. وفي باكستان، توجد تقارير محدودة عن مرض الأكياس المائية. وهذا المرض منتشر في البشر والماشية كذلك، ولكن لم يستكشف بعد. فباكستان بلد زراعي، ونظراً لوضع انتقاله الحيواني المصدر ، هناك حاجة ماسة للبحوث المستقبلية على هذه الأمراض المهملة. ولهذا السبب هذه النسخة المخطوطة الحالية هي محاولة لتسليط الضوء على أهمية مرض الأكياس المائية.

المناقشة: هناك حاجة ماسة للبحوث المستقبلية على مرض الأكياس المائية في باكستان لأنه قد أجريت أعداد قليلة جداً حتى الآن من الأبحاث حول هذا الموضوع. ويسلط انتشار المرض في البلدان المجاورة الضوء على أن باكستان قد تكون في خطر شديد من هذه العدوى الحيوانية المنشأ، وهذا يعزز الحاجة إلى المزيد من الأبحاث. في باكستان تعيش الغالبية العظمى من السكان في المناطق الريفية بوسائل محدودة للمرافق الصحية / الصرف الصحي السليمة. تسهل هذه الظروف تفشي الأمراض مثل داء الأكياس المائية. وبسبب البيانات المتاحة المحدودة، يمكن أن تصبح النتائج أعلى في المستقبل مما يتسبب في تحميل نظام الرعاية الصحية الضعيف أصلاً ما لا يطيق.

فالبلاد لديها ميزانية سنوية ضئيلة للصحة، والتي تنفق على الإصابات المعروفة مثل شلل الأطفال وحمى الضنك والالتهابات الفيروسية الكبدية. هناك حاجة إلى نظام مراقبة مناسب لمرض الأكياس المائية في أنحاء البلاد لأن العلاج عادة ما يكون مكلفاً ومعقداً وقد يتطلب عملية جراحية واسعة و/أو فترات علاج طويلة بالعقاقير. ويتلقى تطوير عقاقير جديدة / مبتكرة وطرق العلاج الأخرى القليل من الاهتمام للغاية، إن وجد. وتشمل برامج توعية التكهنات ضد هذه العدوى التخلص من الديدان من الحيوانات المصابة، وتحسين التفتيش على الأغذية ونظافة المسلخ، وحملات التوعية العامة.

الخلاصة: من المتوقع أن تُظْهِر الجهود البحثية المستقبلية التي تعالج هذه القضايا (البحوث المستقبلية على مرض الأكياس المائية) إذا ما كانت الأوبئة والتشخيص واللقاحات المؤتلفة / الأجسام المضادة المتعلقة بالأكياس المائية يمكن أن تلبي معايير الجودة (النقاء والقوة والسلامة والفعالية) التي حددتها منظمة الصحة العالمية. وينبغي أن يتم إجراء بعض البحوث على الأوبئة والتشخيص المصلي للأكياس المائية في مناطق مختلفة من باكستان والتي قد تكون مفيدة للقضاء الصحيح على داء الأكياس المائية في هذه المنطقة. يجب أن تقوم وزارة الصحة بتنفيذ بعض حملات التوعية للتنبيه في الرأي العام للحد من عبء المرض.

Translated from English version into Arabic by Free bird, through

TRANSLATORS
WITHOUT BORDERS

为什么巴基斯坦需要更多的关于棘球蚴病的研究

Haroon Ahmed, Shahzad Ali, Muhammad Sohail Afzal, Abid Ali Khan, Hamid Raza, Zaheer Hussain Shah, Sami Simsek

摘要

引言：棘球蚴病呈全球分布，在所有有人居住的大陆均有流行。由于近来巴基斯坦多地频繁出现暴发，因此棘球蚴病被描述为一种被忽视的热带病，且被认为是巴基斯坦最被忽视的寄生虫病之一。在流行区，尤其是资源有限的地区，因无法获得合理的治疗，因此有大量的棘球蚴病患者。在巴基斯坦，关于棘球蚴病的报道较少。虽然如此，该病在人和家畜均有流行。巴基斯坦是一个农业国家，加上棘球蚴病传播的人兽共患模式，亟需对其加强研究。这也是本文为什么努力强调棘球蚴病的重要性。
讨论：到目前巴基斯坦对棘球蚴病开展的调查非常少，因此未来亟需加强该病的研究。该病在邻国的流行表明巴基斯坦很可能也受到该病的严重威胁，需要更多的研究以证实。巴基斯坦大部分人生活在农村地区，难以接近合适的卫生设施。这些因素有利于诸如棘球蚴病的暴发。由于数据有限将来可能引起更大的暴发，继而加重本已脆弱的卫生体系的负担。巴基斯坦每年的卫生预算非常有限，而且主要花在脊髓灰质炎、登革和肝炎病毒感染上。需要在巴基斯坦构建合适的棘球蚴病监测系统，因为治疗通常是昂贵的、复杂的，而且常需要大量的手术和（或）长期的药物治疗。研发新的药物和其他治疗手段很少受到重视，即使有的话。避免感染的措施包括对感染动物驱虫、改善食品和屠宰卫生以及教育公众。
结论：期望将来针对棘球蚴病的研究能够阐明棘球蚴病相关的流行病学、诊断和重组的疫苗/抗体能否满足 WHO 要求的质量标准（纯度、效能、安全性和有效性）。需要在巴基斯坦不同地区开展一些棘球蚴病的流行病学和血清学诊断的研究工作，这将有助于该地区消除该病。卫生部门应该采取一些教育行动加强公众的意识，以减少该病的疾病负担。

Translated from English version into Chinese by Qian Men-Bao, edited by Yang Pin, through

CHINESE CENTER FOR DISEASE CONTROL AND PREVENTION
NATIONAL INSTITUTE OF PARASITIC DISEASES

L'urgence de mener des recherches approfondies sur l'échinococcose au Pakistan

Haroon Ahmed, Shahzad Ali, Muhammad Sohail Afzal, Abid Ali Khan, Hamid Raza, Zaheer Hussain Shah, Sami Simsek

RÉSUMÉ

Contexte: L'échinococcose est une maladie que l'on retrouve dans le monde entier surtout sous forme endémique sur tous les continents habités. À cause des multiples accès de la maladie à des endroits différents au Pakistan dans un passé récent, l'échinococcose est vue comme une maladie tropicale négligée et est considérée comme étant l'une des parasitoses les plus négligées du pays. En région endémique, surtout dans celles qui ne disposent pas de suffisamment de ressources, il y a un nombre élevé de patients atteints d'échinococcose dû au manque de traitement adéquat parmi ces communautés. Au Pakistan par exemple, peu d'informations sur l'échinococcose sont disponibles. Cependant, cette maladie attaque l'homme et les animaux d'élevage sans toutefois avoir été étudiée. Le Pakistan est un pays dont l'agriculture constitue l'un des piliers de l'économie et lorsqu'on regarde le mode de transmission de cette maladie de l'animal à l'homme, il y a un besoin alarmant de mener des études sur cette maladie tant négligée. D'où l'importance de cet article qui cherche à mettre en exergue les dangers liés à l'échinococcose.

Discussion: Le besoin de mener une étude sur l'échinococcose au Pakistan est urgent étant donné que très peu d'enquêtes ont été menées jusqu'ici sur ce sujet. Le taux de prévalence de cette maladie dans les pays voisins témoigne à suffisance du risque très grave d'infection zoonotique que court le Pakistan et vient par ailleurs appuyer le besoin de mener des recherches sur cette maladie. Au Pakistan, plus de la moitié de la population vit en milieu rural avec un accès restreint aux ressources hygiéniques/sanitaires adéquates. Cet état des choses favorise l'accès de maladies comme l'échinococcose. Le revers de ce manque de données sur la maladie serait de plus grands accès de cette maladie à l'avenir, ce qui pourrait alourdir le fardeau d'un système de santé déjà fragile.

Le budget annuel attribué à la santé est non seulement maigre, mais il n'est dépensé que pour des infections sur lesquelles on a déjà des informations à l'instar de la polio, la fièvre dengue et les infections à hépatites virales. Un système de surveillance qui s'étend à tout le pays est donc requis pour l'échinococcose puisque le traitement est souvent coûteux, compliqué et pourrait même nécessiter une intervention chirurgical sérieuse et/ou un traitement médicamenteux prolongé. Par ailleurs, le développement de médicaments nouveaux/novateurs et tout autre moyen de traitement ne reçoivent presque pas d'attention. Des propositions de solutions contre cette infection comprennent un déparasitage des bêtes infectées, une meilleure surveillance de la nourriture et de l'hygiène des boucheries, ainsi que l'organisation de campagnes de sensibilisation publics.

Conclusion: Des études futures (des recherches sur l'échinococcose) sur ces questions sont attendues afin déterminer si l'épidémiologie, le diagnostic et les vaccins/anticorps recombinants liés à l'échinococcose peuvent répondre aux normes de qualité (pureté, puissance, sureté et efficacité) définies par l'Organisation mondiale de la Santé. Par ailleurs, des études devront être menées sur l'épidémiologie et le sérodiagnostic de l'échinococcose dans différentes régions du Pakistan qui pourront s'avérer utiles pour l'éradication adéquate de l'échinococcose de cette région. Le ministère de la santé doit mettre en œuvre des campagnes de sensibilisation du grand public afin de réduire les effets de la maladie.

Translated from English version into French by simonyetna, through

Почему нужно уделять больше внимания исследованию эхинококкоза в Пакистане

Харун Ахмед, Шахзад Али, Мухаммед Сохаил Афзаль, Абид Али Хан, Хамид Раза, Захир Хуссейн Шах, Сами Симсек

ТЕЗИСЫ

Исходный контекст. Эхинококкоз распространен по всему миру: эндемические очаги возникают на всех населенных континентах. В недавнем прошлом вспышки эхинококкоза происходили в разных частях Пакистана, однако этому тропическому паразитарному заболеванию здесь уделяют недостаточно внимания. Эндемичные очаги обычно возникают в бедных регионах. В них живет много больных эхинококкозом, поскольку в таких общинах

недоступно правильное лечение. Лишь небольшое количество сообщений о случаях заболевания эхинококкозом регистрируется в Пакистане. Болезнь пока должным образом не изучена. Она поражает как людей, так и домашних животных. Пакистан — аграрная страна, и из-за зоонозного характера передачи инфекции существует необходимость в ее дальнейшем изучении. Настоящая работа представляет собой попытку привлечь больше внимания к проблеме эхинококкоза.

Анализ. На данный момент было проведено лишь небольшое количество исследований эхинококкоза. Это заболевание распространено в соседних странах, что лишь подчеркивает риск проникновения этой зоонозной инфекции в Пакистан. Большинство населения Пакистана живет в сельской местности с ограниченным доступом к надлежащим гигиеническим и санитарно-бытовым условиям, что способствует возникновению вспышек эхинококкоза. Данных о заболевании мало, поэтому в будущем велик риск возникновения еще более крупных очагов, из-за чего и без того слабая система здравоохранения страны может быть перегружена.

Бюджет здравоохранения Пакистана очень мал. В основном он расходуется на борьбу с распространенными заболеваниями, такими как полиомиелит, лихорадка денге и вирусные инфекций печени. Нужно создать надлежащую систему надзора за эхинококкозом на всей территории страны, так как обычно лечение болезни стоит дорого, оно сложное и может потребовать обширного хирургического вмешательства и/или длительной лекарственной терапии. Мало внимания уделяется разработке новых лекарственных препаратов и других методов лечения. Профилактика эхинококкоза предполагает дегельминтизацию зараженных животных, улучшение гигиены при их забое, тщательный осмотр продуктов питания и проведение просветительских кампаний.

Заключение. Ожидается, что будущие исследования эхинококкоза покажут, насколько существующие рекомбинантные вакцины/антитела и методики эпидемиологии и диагностики, направленные на борьбу с этим заболеванием, соответствуют стандартам качества (требованиям к чистоте, эффективности и безопасности) Всемирной организации здравоохранения. Чтобы искоренить эхинококкоз в различных регионах Пакистана, нужно провести работу в области эпидемиологии и серодиагностики. Министерство здравоохранения должно провести информационно-просветительские кампании, направленные на информирование населения и борьбу с заболеванием.

Translated from English version into Russian by Vadim Frolenko, through

TRANSLATORS
WITHOUT BORDERS

Por qué se debe investigar más sobre la equinococosis en Pakistán

Haroon Ahmed, Shahzad Ali, Muhammad Sohail Afzal, Abid Ali Khan, Hamid Raza, Zaheer Hussain Shah, Sami Simsek

RESUMEN

Introducción: La equinococosis tiene una distribución geográfica mundial, con focos epidémicos en cada continente habitado. Debido a los frecuentes brotes en distintas partes de Pakistán en los últimos años, la equinococosis se considera una enfermedad tropical desatendida y una de las enfermedades parasitarias más descuidadas del país. En zonas de epidemia, principalmente en lugares con recursos limitados, hay un gran número de pacientes con equinococosis, ya que estas comunidades no tienen acceso a un tratamiento adecuado. En Pakistán, existen pocos informes sobre la equinococosis. Esta enfermedad es prevalente en los seres humanos y también en el ganado, aunque todavía no se la ha investigado. Pakistán es un país agrícola y, debido al modo de transmisión zoonótica de la enfermedad, existe una necesidad imperiosa de investigación a futuro sobre esta enfermedad desatendida. Es por esto que el presente manuscrito tiene como objetivo resaltar la importancia de la equinococosis.

Debate: Existe una necesidad imperiosa de investigación a futuro sobre la equinococosis en Pakistán, ya que a la fecha existen muy pocas investigaciones sobre este tema. La prevalencia de esta enfermedad en países vecinos resalta el hecho de que Pakistán podría tener un alto riesgo de esta infección zoonótica y avala la necesidad de más investigación. En Pakistán, la mayor parte de la población vive en zonas rurales y tiene acceso limitado a instalaciones sanitarias y de higiene adecuadas. Estas condiciones fomentan los brotes de enfermedades como la equinococosis. Debido a la escasa información disponible, podrían suceder mayores brotes en el futuro, causando un colapso en el ya débil sistema de salud.

El país tiene un presupuesto anual muy escaso para la salud, el cual se gasta en infecciones conocidas como la polio, el dengue y en infecciones virales hepáticas. Se requiere un sistema de control adecuado para la equinococosis en todo el país, ya que el tratamiento suele ser caro, complicado y puede requerir una cirugía extensa o una terapia farmacológica prolongada. El desarrollo de nuevas drogas y de otras modalidades de tratamientos recibe muy poca, si es que alguna, atención. Los programas de concientización de pronósticos para combatir esta infección incluyen la desparasitación de animales infectados, una mejor inspección de los alimentos y de la higiene en los mataderos, y campañas de educación pública.

Conclusión: Para demostrar si la epidemiología, el diagnóstico y las vacunas/anticuerpos recombinantes relacionados con la equinococosis pueden cumplir con los estándares de calidad (pureza, potencia, seguridad y eficacia) que estipula la Organización Mundial de la Salud, se deben realizar esfuerzos de investigaciones a futuro que traten estos temas (investigaciones a futuro sobre la equinococosis). Se deberían llevar a cabo algunos trabajos de investigación sobre la epidemiología y el diagnóstico serológico de equinococosis en diferentes zonas de Pakistán que pudieran ser de utilidad para una erradicación adecuada de la equinococosis en esta región. El departamento de salud debería implementar algunas campañas de concientización para el público en general para así reducir la carga de morbilidad.

Translated from English version into Spanish by Adrián Rueda, through

TRANSLATORS
WITHOUT BORDERS
